# Neuroscience researches at Belyaev conference-2017

**DOI:** 10.1186/s12868-018-0410-7

**Published:** 2018-04-19

**Authors:** Yuriy L. Orlov, Leonid L. Moroz, Ancha V. Baranova

**Affiliations:** 1 Novosibirsk State University, Novosibirsk, Russia; 2 The A.O. Kovalevsky Institute of Marine Biological Research of RAS, Sevastopol, Russia; 3 University of Florida, Gainesville, FL USA; 4 Research Centre of Medical Genetics, Moscow, Russia; 5 Moscow Institute of Physics and Technology (State University), Dolgoprudnyi, Russia

This thematic issue of *BMC Neuroscience* continues the series of special post-conference journal issues published by BioMed Central. These Special Issues present materials from the bioinformatics and systems biology summit BGRS\SB (Bioinformatics of Genome Regulation and Structure\Systems Biology) held in Novosibirsk, Russia in August 2017. Here we present the works on neurobiology and computer genomics discussed in the Neuroscience section of the Belyaev Conference (http://conf.bionet.nsc.ru/belyaev100/en). This Special Issue is accompanied by the other Special Issues collecting the works in the fields of evolutionary biology, plant biology, and genetics, which were published as *BMC Evol Biol* [1], and *BMC Genetics* [2] supplements. The Belyaev Conference-2017 was a memorial event devoted to the 100th anniversary of Academician Dmitri K. Belyaev. Evolutionary aspects of Belyaev’s own works were described in [1, 3] and in a Special Issue of *BMC Evol Biol* (https://bmcevolbiol.biomedcentral.com/articles/supplements/volume-17-supplement-2).

The Belyaev conference-2017 continued the tradition of BGRS\SB by keeping several thematic sections which were run simultaneously. Previously published special issues of *BMC Genomics*, *BMC Genetics*, and *BMC Evolutionary Biology* covered the proceeding of the BGRS\SB-2016 conference and SBB-2016 School in Novosibirsk [4–6] as well as earlier BGRS\SB-2014 meeting (https://bmcgenomics.biomedcentral.com/articles/supplements/volume-15-supplement-12).

Both experimental works and the reviews on various current topics in neuroscience 2017 are put together in *BMC Neuroscience* after being presented for attendants of the Belyaev conference-2017.

The work by Polesskaya et al. [7] reviews optogenetic constructs that employ photosensitive proteins to transduce the signal and regulate gene transcription within specific sets of neurons. Optogenetics has become widely recognized for its success in real-time control of brain activity by utilizing non-mammalian photosensitive gating proteins. This type of transcriptional regulation could potentially be used to produce biological drugs in situ or on demand, by repeatedly applying light to the tissue.

Tikhonova et al. [8] discuss modulation of the expression of genes related to the metabolism of amyloid-beta. The prevailing “amyloid cascade” hypothesis about the etiology of Alzheimer’s disease (AD) posit that modulating the metabolism of amyloid-beta (Aβ) could be an effective strategy for the prevention and therapy of AD. An antibiotic ceftriaxone, which possesses neuroprotective activity, also reduces both the cognitive deficits and the extent of neurodegenerative changes in OXYS rats, a recognized model for the sporadic AD. Comparison of the gene expression in tissues of untreated OXYS and Wistar rats showed a significant decrease in mRNA levels of *Ace2* in the frontal cortex and hypothalamus, and of *Actb* in the amygdala and points at novel targets of ceftriaxone action.

Babenko et al. [9] continues the topic of Alzheimer’s disease. They present haplotype analysis of ApoE SNPs in a cohort of patients enrolled in Alzheimer Disease Neuroimaging Initiative (ADNI) and in HapMap Asian, African and European descent populations. It was found that the Asian population is the most distinct one for ApoE haplotype, with the T allele variant at AD-associated SNP rs429358 being expanded as a protective allele during the populations divergence possibly due to the predominantly meat diet not common for primates. Haplotypic inference revealed an enrichment in protective alleles, including intronic SNP rs769449, in European sample. The results could be utilized for the risk assessment based on ethnic descent of an individual.

Bryzgalov et al. [10] analyzed a set of novel functional variants at the GWAS-implicated loci implicated in major depressive disorder, bipolar affective disorder and schizophrenia for their role in cognitive functions. The authors focused on regulatory genomic regions and provided important insights about fourteen regulatory SNPs located within these loci, with six of the variants unreported previously.

Follow-on series of related works in the areas of genomics, genetics discussed at “Belyaev conference-2017” and other related meetings in Novosibirsk are published in the Special Issues of BMC Evolutionary Biology (https://bmcevolbiol.biomedcentral.com/articles/supplements/volume-17-supplement-2), *BMC Genetics* (https://bmcgenet.biomedcentral.com/articles/supplements/volume-18-supplement-1, published at the end of 2017), *BMC Genomics* (https://bmcgenomics.biomedcentral.com/articles/supplements/volume-19-supplement-3), *BMC Medical Genomics* (https://bmcmedgenomics.biomedcentral.com/articles/supplements/volume-11-supplement-1), *BMC Structural Biology* (https://bmcstructbiol.biomedcentral.com/articles/supplements/volume-18-supplement-1) (published in parallel to this issue in 2018).

The readers are welcome to visit Novosibirsk in August 2018 for XI-th BGRS\SB-2018 conference (http://conf.bionet.nsc.ru/bgrssb2018/en/) and neurobiology sessions.
